# Detection of Mutations in *pncA* in *Mycobacterium tuberculosis* Clinical Isolates from Nepal in Association with Pyrazinamide Resistance

**DOI:** 10.3390/cimb44090283

**Published:** 2022-09-08

**Authors:** Dipti Shrestha, Bhagwan Maharjan, Jeewan Thapa, Mwangala Lonah Akapelwa, Precious Bwalya, Joseph Yamweka Chizimu, Chie Nakajima, Yasuhiko Suzuki

**Affiliations:** 1 Division of Bioresources, Hokkaido University International Institute for Zoonosis Control, Kita 20, Nishi 10, Kita-ku, Sapporo 001-0020, Japan; 2 Department of Microbiology, Kathmandu College of Science and Technology, Tribhuvan University, Kathmandu 44600, Nepal; 3 German Nepal Tuberculosis Project c/o Nepal Anti-Tuberculosis Association, Kalimati, Kathmandu 44600, Nepal; 4 National Tuberculosis Control Center, Thimi, Bhaktapur 44800, Nepal; 5 International Collaboration Unit, Hokkaido University Research Center for Zoonosis Control, Kita 20, Nishi 10, Kita-ku, Sapporo 001-0020, Japan

**Keywords:** pyrazinamide, *pncA*, URR, mutation, *Mycobacterium tuberculosis*

## Abstract

Without the proper information on pyrazinamide (PZA) susceptibility of *Mycobacterium tuberculosis* (MTB), PZA is inappropriately recommended for the treatment of both susceptible and multidrug-resistant tuberculosis (MDR-TB) in Nepal. This study aimed to collect information regarding PZA susceptibility in MTB isolates from Nepal by analyzing *pncA* and its upstream regulatory region (URR). A total of 211 MTB isolates were included in this study. Sequence analysis of *pncA* and its URR was performed to assess PZA resistance. First-line drug susceptibility testing, spoligotyping, and sequence analysis of *rpoB*, *katG*, the *inhA* regulatory region, *gyrA*, *gyrB*, and *rrs* were performed to assess their association with pncA mutation. Sequencing results reveal that 125 (59.2%) isolates harbored alterations in *pncA* and its URR. A total of 57 different mutation types (46 reported and 11 novel) were scattered throughout the whole length of the *pncA* gene. Eighty-seven isolates (41.2%) harbored mutations in *pncA*, causing PZA resistance in MTB. There was a more significant association of *pncA* alterations in MDR/pre-extensively drug-resistant (Pre-XDR) TB than in mono-resistant/pan-susceptible TB (*p* < 0.005). This first report on the increasing level of PZA resistance in DR-TB in Nepal highlights the importance of PZA susceptibility testing before DR-TB treatment.

## 1. Introduction

Tuberculosis (TB), caused by *Mycobacterium tuberculosis* (MTB), is one of the leading health problems and causes of death worldwide. In 2019, an estimated 10.0 million people suffered from TB, and 1.4 million people died of TB [1]. The increasing level of drug-resistant TB (DR-TB) each year challenges the effective management of TB. In 2019, the World Health Organization (WHO) estimated half a million new cases of rifampicin-resistant TB (RR-TB), with 78% of multidrug-resistant TB (MDR-TB). About 8.5% of MDR-TB cases were assumed to be extensively drug-resistant tuberculosis (XDR-TB) [1]. The estimated burden of annual DR-TB in Nepal is around 1500 cases, with around 350 to 450 MDR-TB cases [2].

Pyrazinamide (PZA) is an important first-line anti-TB drug with sterilizing activity against non-replicating persistent bacilli, which is used at the initial intensive phase of chemotherapy in combination with other first-line drugs: isoniazid (INH), rifampicin (RIF), and ethambutol (EMB). PZA shortens the treatment regimen for both drug-susceptible and drug-resistant TB [1].

Despite the important role of PZA in TB treatment, PZA resistance in MTB has increased in both susceptible and MDR-TB cases. The estimated global burden of new PZA-resistant TB cases annually is 1.4 million cases, of which 270,000 cases occur in MDR-TB patients [3].

PZA is an indispensable drug included in all TB treatment regimens; therefore, the accurate understanding of PZA susceptibility and the monitoring of the spread of drug-resistant strains is important for the timely management of TB. However, phenotypic PZA susceptibility testing (PST) is difficult to perform, has a long turnaround time, and is not cost-effective for a developing country such as Nepal. Additionally, the requirement of an acidic medium has made phenotypic PST unreliable [4,5]. As a result, phenotypic PST is not routinely performed in most laboratories worldwide, including Nepal.

PZA is a pro-drug which enters the bacteria through passive diffusion and is converted into its active form, pyrazinoic acid (POA), by the non-essential enzyme pyrazinamidase (PZase), encoded by the *pncA* [6,7]. POA disrupts the bacterial membrane energetics and inhibits the membrane transport function in an acidic environment [8]. Mutation in *pncA* and its upstream regulatory region (URR), leading to a loss of PZase activity, is the major mechanism for PZA resistance in MTB and has been observed in 45 to 85% of PZA-resistant MTB isolates [9,10,11]. Thus, sequencing of *pncA* can rapidly and effectively judge PZA susceptibility, indicating its usefulness in the development of rapid molecular tools to detect PZA susceptibility in MTB.

Since both phenotypic and genotypic PST are currently unavailable, the prevalence of PZA resistance in MTB is unknown in Nepal. The lack of proper information on PZA susceptibility has resulted in the inappropriate recommendation of PZA for chemotherapy in both susceptible and MDR-TB. Therefore, we aimed to elucidate PZA susceptibility in MTB isolates from Nepal by analyzing *pncA*.

## 2. Materials and Methods

### 2.1. Sample Collection and Study Sample

A total of 211 samples collected at the German Nepal Tuberculosis Project (GENETUP), Nepal, from August 2008 to February 2011 were included in this study. These samples were collected from 155 males and 56 females, with an age range from 12 to 82 years.

The sputum samples used in this investigation were decontaminated for 15 min in a 50 mL Falcon tube with 4 percent NALC-NaOH, neutralized for the same amount of time with phosphate buffer (pH 6.8), and then centrifuged at 3000× *g* for 20 min. After centrifugation, the supernatant was discarded, and 1 mL of phosphate buffer was used to resuspend the sediment. Two slants of Löwenstein–Jensen (LJ) media were infected with resuspended samples (200 uL each) (BBLTM MycobactoselTM LJ Medium; Becton, Dickinson and Company, Sparks, MD, USA).The samples that had been resuspended were utilized to make smears. The inoculated LJ slants were kept at 37 °C for 24 h while mycobacterial growth was observed. The TB Ag MPT64 Rapid test (SD Standard Diagnostics) was used to distinguish MTBC from non-tuberculous mycobacteria (NTM) in mycobacteria-positive cultures [11,12,13,14,15,16].

### 2.2. Drug Susceptibility Testing (DST)

DST for INH, RIF, EMB (FatolArzneimittel GmbH, Schiffweiler, Germany), and streptomycin (STR) (Sigma-Aldrich, St. Louis, MO, USA)was performed using the indirect proportion method on the LJ medium, with a critical concentration of 0.2, 40, 2, and 4 ug/mL, respectively [12]. Briefly, a 1.0 McFarland standard mycobacterial suspension was prepared from a freshly grown colony on LJ medium and serially diluted from 10^−1^ to 10^−4,^ and then inoculated onto LJ slants with and without drugs and incubated at 37 °C for 28 to 42 days. Interpretation of results was performed according to the growth of bacilli on two slants. An isolate was determined to be resistant to a drug if it had ≥1% colony growth on a drug-containing medium when compared with a control isolate.

### 2.3. DNA Extraction

Hain DNA was processed for PCR using the Genotype MTBDRplusVer 2 (HainLifescience, Nehren, Germany) according to the manufacturer’s instructions. Colonies from successful cultures were kept suspended in 300 µL of DNA-free distilled water for 20 min at 95 °C. The heated sample was centrifuged for 5 min at 10,000× *g* after 15 min in an ultrasonic water bath. The DNAs of 211 isolates were utilized for PCR amplification [13,14,15].

### 2.4. Spoligotyping

The genotypes of the MTB isolates were identified using spoligotyping, as described earlier [13]. Briefly, a primer set was used to amplify the direct repeat region (Table 1) [14,15], and the resulting PCR products were hybridized to a set of 43 spacer-specific oligonucleotide probes on the membrane. Hybridization patterns were converted into binary and octal formats to determine the Spoligo-international types (SITs). The resulting SITs were compared with the patterns previously reported in the SpolDB4 database (http://www.pasteurguadeloupe.fr:8081/SITVIT_ONLINE/ (accessed on 10 March 2015)).

### 2.5. PCR Amplification and DNA Sequencing

Targeted gene fragments (*rpoB*, *katG*, *inhA* regulatory region, *gyrA*, *rrs*, and *pncA* with its URR) were amplified and sequenced using primers as previously described (Table 1) [14,15,16]. PCR was performed in a 20 μL reaction mixture consisting of 5X Green GoTaq^®^ Reaction Buffer (Promega Corp, Madison, WI, USA), 0.5 M betaine, 0.25 mM MgCl_2_, dNTPs (0.25 mM each), primers (0.5 μM each), 1.25 U GoTaq^®^ DNA Polymerase (Promega Corp), and 1 μL of template DNA (approximately 10 ng/μL). There was one cycle of initial denaturation at 95 °C for 10 s annealing at 50 or 55 °C for 10 s, an extension at 72 °C for 30 s, and a final extension at 72 °C for 5 min. Upon amplification, PCR products were confirmed by agarose gel electrophoresis. PCR products were purified using ExoSAP (Thermo Fisher Scientific, Inc., Waltham, MA, USA) and sequenced using a 3500 Genetic Analyzer (Thermo Fisher Scientific, Inc.) using both forward and reverse primers. The obtained sequences were aligned with the corresponding sequences of the wild-type MTB H37Rv using Bio-Edit software (version 7.0.9).

### 2.6. SUSPECT-PZA

The SUSPECT-PZA (http://biosig.unimelb.edu.au/suspect_pza/ (accessed on 5 October 2019)) is a web-server predictive tool to rapidly predict the PZA susceptibility using computational assessment of a single-nucleotide substitution. This web tool only uses the single-nucleotide substitution at the putative *pncA* region to predict the PZA susceptibility of MTB. The 3D structure of *pncA* as an output of the single-nucleotide substitution from this web tool could accurately predict mutations associated with PZA resistance [17].

### 2.7. Statistical Analysis

All data were analyzed using IBM SPSS Statistics for Windows software version 22.0 (IBM Corp., Armonk, NY, USA). A two-tailed Fisher’s exact test was used to compare the PZA resistance-associated mutations with the resistance-conferring mutations for other drugs, including STR, INH, RIF, and EMB. The odds ratio was calculated to detect the association between the frequency of *pncA* mutations and that of *rpoB* or *katG* or *inhA* mutations, *gyrA* or *gyrB* or *rrs* mutations, and *pncA* mutations among different drug-resistant profiles and MTB genotypes. A *p*-value of less than 0.05 was considered statistically significant.

## 3. Results

### 3.1. MTB Genotypes and Drug Resistance Patterns

Using spoligotyping, 211 MTB isolates were identified as lineage 1 (*n* = 13), 2 (*n* = 113), 3 (*n* = 56), and 4 (*n* = 29), as shown in Table 2. The DST results show that a majority of isolates were MDR (108, 51.2%), followed by Pre-XDR isolates (57, 27%), pan-susceptible (41, 19.4%), and mono-resistant (5, 2.4%) as shown in Table 2. Eight different first-line drug susceptibility patterns among MTB isolates were observed (Supplementary Table S1). The drug susceptibility patterns for INH, RIF, EMB, and STR were available for 201 isolates, but only INH and RIF susceptibility results were available for 10 isolates (Supplementary Table S1).

### 3.2. pncA Mutation Profile

Sequencing results reveal that 125 (59.2%) isolates harbored alterations in *pncA* and/or its URR. A total of 57 different types of alterations (46 reported and 11 novel) scattered through the *pncA* were found. Three alterations in URR, single-nucleotide substitutions (A-11C and A-11G) in three isolates, and an insertion of C between -2 and -3 in one isolate were observed. Among 66 isolates with single-nucleotide substitutions, 38 types in 63 isolates were associated with phenotypic PZA resistance. Insertion and deletion of nucleotides in *pncA* causing frameshift associated with PZA resistance were observed in 11 isolates. No amplification of *pncA* was found in four isolates. The most frequent *pncA* mutation observed was associated with Leu182Ser in 12 isolates, followed by Leu4Ser in 6 isolates. Two types of silent mutations (Ser65Ser and Thr153Thr) were found in this study. Silent mutation Ser65Ser was significantly associated with the CAS genotype (48/56). Mutations associated with Thr100Pro and Val157Gly do not show any concomitant results between SUSPECT-PZA and the literature review (Supplementary Table S2).

Eighty-seven isolates (41.2%) had mutations in *pncA* and its URR (Table 3). The *pncA* mutations were found in 1 (2.1%) mono-resistant, 48 (44.4%) MDR, and 38 (66.6%) XDR-MTB isolates (Table 4). There was a more significant association of *pncA* mutations with MDR/Pre-XDR-TB than with pan-susceptible/mono-resistant MTB (*p* < 0.005). Among 87 isolates with *pncA* mutations, the majority were observed in lineage 2 (47, 54%), followed by lineage 4 (18, 20.6%), 3 (15, 17.2%), and 1 (7, 8%) (Table 5).

The distribution of *pncA* mutation frequency among different age-wise participants showed a wide range from 0 to 76.2%. The highest *pncA* mutation frequency (76.2%) was observed among the 16–20 age group of participants (Supplementary Table S3).

### 3.3. Association of pncA Mutations with rpoB, katG, inhA, gyrA, gyrB, and rrs Mutations

Among 108 MDR isolates, 45 (48.3%) had mutations in URR or the coding region of *pncA*, while 3 (20.0%) isolates without *rpoB* or *katG* or *inhA* mutation had mutations in *pncA* coding region. Mutations in coding region of *pncA* were 3.75 times more likely to occur in *rpoB* or *katG* or *inhA* mutants than in *rpoB* or *katG* or *inhA* non-mutants (*p* = 0.05) (Table 6). Similarly, a higher frequency of *pncA* mutations was found in 34 (77.3%) Pre-XDR isolates than in isolates without *gyrA* or *gyrB* or *rrs* mutants (Odds ratio = 7.65, *p* = 0.003) (Table 6).

## 4. Discussion

To the best of our knowledge, this study is the first attempt at molecular analysis of PZA susceptibility of MTB isolates in Nepal. Alterations in *pncA* and/or its URR were found in about 41.2% of MTB isolates. They scattered throughout the *pncA* with different mutations at codons, as previously described [18]. Other studies reported PZA resistance percentages among MTB isolates as 24.4% in Thailand [8], 32.3%, 43.1%, or 61.2% in China [19,20,21], 45% in Bangladesh [19], 52.3% in India [20], and 61.2% in Japan [21]. Our study shows values within the range of other countries, which suggests that PZA resistance could be predicted through *pncA* sequencing.

Three Pre-XDR isolates with single-nucleotide substitutions (A-C and A-G) at the URR-11 position and one isolate with an insertion of C between -2 and -3 were found in this study. Since the nucleotide position at URR-11 is a part of the Shine–Dalgarno sequence, [22] the substitution from A to C or G may cause low affinity of mRNA to the 3′ end of 16 ribosomal RNAs, and hence, it is associated with reduced expression of PncA. Therefore, the two single-nucleotide substitutions at URR A-11C and A-11G were associated with PZA resistance.

In this study, the most frequent mutation, T545C (Leu182Ser), was found in 12 (5.6%) isolates, followed by T11C (Leu4Ser) in 6 (2.8%) isolates. It was reported to cause PZA resistance [11,23] and showed identical similar mutations in *rpoB*, *katG*, *gyrA*, or *rrs*, suggesting the spread of those mutant strains in an outbreak. The 12-loci MIRU-VNTR patterns of three Beijing strains with T545C (Leu182Ser) were confirmed to be similar, which suggests the involvement of these mutants in an outbreak.

The mutation C169G (His57Asp) was found in two isolates. This mutation is a unique mutation specific to *M. bovis* strains, including BCG substrains (Pasteur, Copenhagen, Glaxo, Tokyo, Tice, and Phipps), which are naturally resistant to PZA [23]. However, the two isolates in this study were confirmed to be MTB using spoligotyping. PZA-resistant MTB isolates harboring the same mutation were reported earlier [6,24,25]. The MTB strain harboring a change in His57 to Asp57 might arise with a negative PZase activity and could eventually spread widely around the world.

Forty-one types of mutations (excluding silent mutation) that are associated with PZA resistance were found in this study. Mutations in *pncA* were dispersed throughout the gene, and some degree of clustering of mutations was found in three regions (amino acid 3–17, 61–85, and 127–154) of the PncA among PZA-resistant isolates, as has been widely reported [7,8,15,20,21,26,27,28]. The diverse and scattered nature of *pncA* mutations at different locations, as described by previous studies, poses a challenge for developing a rapid molecular assay for the detection of PZA susceptibility. Isolates harboring mutations at active site residues or metal ion binding site residues causes the loss of enzymatic activity or the chelation of ion atoms [23,24,29], leading to the emergence of PZA resistance. This information could be used to predict PZA resistance using molecular PST. Mutations Ser59Phe, Val139Ala, and Leu116Pro have been previously reported in PZA-susceptible and resistant strains. This discrepant result might be due to the inaccuracy of the phenotypic PZA susceptibility found using the MGIT 960 method [4,5] or due to the presence of hetero-resistance or a mixed bacterial population. These mutations were compared with the PZA susceptibility using SUSPECT-PZA and interpreted. Ser59Phe, Val139Ala, and Leu116Pro were interpreted as resistant, resistant, and susceptible, respectively.

No amplification of *pncA* was observed in one pan-susceptible and three Pre-XDR MTB isolates, as in previous reports [21,25]. The possible reason for no amplification of *pncA* might be changes at the primer binding site(s) or an entire *pncA* deletion. The deletion of an entire *pncA* causes negative PZase and leads to PZA resistance. These four *pncA* mutants show good PCR and sequencing results for *rpoB*, *katG*, *inhA*, *gyrA*, *gyrB*, and *rrs* genes. So, we could deny the possibility of technical failure of *pncA* PCR. However, to confirm the whole length of *pncA* deletion in MTB, PCR using primers targeting distinct sites should be performed.

In total, 87 (41.2%) isolates with *pncA* mutations were associated with PZA resistance. PncA is the non-essential enzyme, and the mutations in *pncA* or the deletion of an entire *pncA* have no fitness cost in bacteria [17,29]. Since PZA is included in the treatment of non-DR and DR-TB without the phenotypic DST in Nepal, this practice causes the selective pressure of PZA during MTB infection. Our study shows high variation in mutation frequency at the *pncA* nucleotide in MTB. This phenomenon has been reported by in vivo studies, which have shown that selective pressure occurs in the presence of PZA, causing maximal variation in the average mutation frequency at the *pncA* nucleotide [29].

We did not find *pncA* mutations in pan-susceptible and mono-resistant isolates in our study, except for one isolate with negative *pncA* PCR results. Most of the *pncA* mutations associated with PZA resistance were found among MDR-TB (44.4%) and Pre-XDR -TB (66.6%) isolates. The statistical analysis showed that *pncA* alterations were more likely to occur among MDR and Pre-XDR-TB isolates than pan-susceptible/mono-resistant TB isolates. Other studies have reported a high prevalence of PZA resistance among MDR and Pre-XDR/XDR-TB isolates [18,26]. An explanation for this could be that bacterial exposure to antibiotics (RIF, fluoroquinolones, and aminoglycosides) can generate reactive oxygen species that induce the SOS system, which increases mutation frequency and facilitates the development of additional drug resistance [29]. The increasing rate of PZA resistance among DR-TB highlights the need for the prudent use of PZA for drug-resistant TB. In addition, *pncA* mutation was 3.75 and 7.65 times more likely to occur in *rpoB* or *katG* or *inhA* mutants and *gyrA* or *gyrB* or *rrs* mutants, respectively. Our results suggest that the detection of *pncA* mutations could be useful in the prediction of the prevalence of PZA resistance among MDR and Pre-XDR-TB isolates in Nepal. Furthermore, this information could indicate the proportions of PZA resistance among the different categories of MTB isolates from Nepal.

A wide range of *pncA* mutation frequencies were observed among different age groups. Even though there seems to be a wide range of *pncA* mutation frequency among different age groups, the distribution of participants according to age (5-year difference) was not uniform, resulting in inconsistent frequency distributions. The frequency of PZA resistance among lineage 2 MTB isolates was slightly higher than in other lineage isolates. Due to uneven sample size, PZA susceptibility did not correlate with the genotypic lineage of MTB, as previously reported [4]. The silent mutation Ser65Ser was linked to the PZA-susceptible MTB isolates of lineage, in agreement with the other literature [30].

A limitation of our study is the lack of phenotypic PZA susceptibility testing data for the isolates. Nonetheless, most of the alterations in *pncA* were correlated with in vitro/in vivo susceptibility studies and phenotypic susceptibility in the literature as well as in the SUSPECT-PZA web tool, which enabled us to estimate the potential prevalence of PZA resistance [17,29,31]. Even though SUSPECT-PZA can only be used for single-nucleotide polymorphisms at putative *pncA*, it has a positive predictive value of 95.4%; hence, it is a reliable and convenient alternative to MGIT 960, the existing gold-standard method [16]. Moreover, resistance to PZA has also been associated with mutations in other genes, including *rpsA*, *panD*, and others [32,33]. Therefore, it is important to also analyze these genes when considering developing a rapid diagnostic tool for the accurate prediction of PZA resistance in MTB.

In conclusion, the rate of *pncA* mutation was high in MDR-TB/Pre-XDR-TB in our study, and most of the *pncA* mutations resulted in PZA resistance in MTB. This increased number of PZA resistance cases among DR-TB in Nepal is alarming and necessitates routine testing for PZA susceptibility. The increasing rate of PZA resistance in drug-resistant TB has reduced the value of PZA for DR-TB treatment so much that PZA is now categorized in Group C according to the updated WHO consolidated treatment guidelines for drug-resistant tuberculosis [1]. Nevertheless, PZA is still used for both drug-susceptible and drug-resistant TB. This emphasizes the importance of PZA in TB treatment following PZA susceptibility testing. Considering the high cost and long turnaround time of phenotypic DST in developing countries, including Nepal, we recommend the more feasible method of molecular *pncA* sequencing for the detection of PZA susceptibility. Our work emphasizes the value of PZA susceptibility testing before its use in the treatment of DR-TB globally, Nepal included. Based on analysis of the results of our investigations using Nepali samples and SUSPECT-PZA, it was determined that MTB isolates had a greater level of PZA resistance, thus highlighting the importance of performing phenotypic and molecular PZA susceptibility testing prior to administering PZA for DR-TB treatment.

## Figures and Tables

**Table 1:** Primers used for PCR amplification and sequencing of targeted genes in *Mycobacterium tuberculosis*.

Locus	Primers	Nucleotide Sequence (5′-3′)	Product Size (bp)
*rpoB*	ON-513 (Forward) ON-914 (Reverse)	CAGGACGTGGAGGCGATCAC GAGCCGATCAGACCGATGTTGG	278 bp
*katG*	ON-114 (Forward) ON-103R (Reverse)	ATGGCCATGAACGACGTCGAAAC CGCAGCGAGAGGTCAGTGGCCAG	392 bp
*inhA*	ON-097 (Forward) ON-098 (Reverse)	TCACACCGACAAACGTCACGAGC AGCCAGCCGCTGTGCGATCGCCA	231 bp
*gyrA*	ON-747 (Forward) ON-748 (Reverse)	AGCGCAGCTACATCGACTATGCG CTTCGGTGTACCTCATCGCCGCC	321 bp
*rrs*	ON-1066 (Forward) ON-1067 (Reverse)	CGGATCGGGGTCTGCAACTCGAC CAAGAACCCCTCACGGCCTACG	299 bp
*pncA*	ON-1464 (Forward) ON-1465 (Reverse)	GCACCAAGGCCGCGATGACAC CGCGCGTCACCGGTGAACAACC	561 bp
direct repeat region	DRa (Forward) DR-R (Reverse)	GGTTTTGGGTCTGACGAC CCGAGAGGGGACGGAAAC	Varios

**Table 2:** Distribution of MTB isolates according to genotypes and drug resistance patterns.

Types of Mutations	No. of Mutation Types	No. of Isolates
Nucleotide substitution	45	109
URR	2	3
Amino acid substitution	39	66
Termination	2	2
Silent mutation	2	38
Nucleotide deletion	4	4
Nucleotide insertion	6	6
URR	1	1
Putative region	5	5
Nucleotide substitution + deletion	1	2
No amplification	1	4
Total	54	125

**Table 3:** Distribution of polymorphisms of *pncA* among 211 *Mycobacterium tuberculosis* isolates from Nepal.

Lineages	Pan-Susceptible	Mono-Resistant	MDR	Pre-XDR	Total
**1**	3	0	8	2	13
**2**	12	3	62	36	113
**3**	19	2	23	12	56
**4**	7	0	15	7	29
**Total**	41	5	108	57	211

**Table 4:** Mutation profile among different drug resistances of MTB isolates.

Genotype	Pan-Susceptible/ Mono-Resistant	MDR	Pre-XDR	Total
Alteration in *pncA* and its URR	1	48	38	87
WT	45	60	19	124
Total	46	108	57	211

**Table 5:** Mutation profile among MTB lineages.

Mutation Patterns	Lineages				Total
	1	2	3	4	
Mutations in *pncA* and its URR	7	47	15	18	87
WT	6	66	41	11	124
Total	13	113	56	29	211

**Table 6:** Comparison of *pncA* mutation frequencies with *rpoB*, *katG*, and *inhA* genes in 108 MDR-TB isolates and *gyrA*, *gyrB*, and *rrs* genes in 57 Pre-XDR-TB isolates.

Mutation Patterns	No. of Isolates		Odds Ratio, 95% CI	*p*-Value
	With *pncA* Mutations	Without *pncA* Mutations		
***rpoB* or *katG* or *inhA* mutation**	45	48	3.75 (0.9 to 14.1)	0.05
None in above	3	12		
***gyrA* or *gyrB* or *rrs* mutation**	34	10	7.65 (1.9 to 30.18)	0.003
None in above	4	9		

## Data Availability

The data that support the findings of this study are available from the corresponding author, Y.S., upon reasonable request.

## Supplementary Materials

The following supporting information can be downloaded https://www.mdpi.com/article/10.3390/cimb44090283/s1. Table S1: Distribution of first-line drug-susceptibility patterns in MTB lineages; Table S2: Mutations found in the *pncA* gene and its upstream regulatory region from *M. tuberculosis* isolates from Nepal [34]; and Table S3: Distribution of *pncA* mutation frequency among different age-wise participants.
