# Supplementary material for: Detection of Mutations in pncA in Mycobacterium tuberculosis Clinical Isolates from Nepal in Association with Pyrazinamide Resistance
Source: Curr Issues Mol Biol. 2022 Sep 8;44(9):4132–41. doi: 10.3390/cimb44090283 (PMC9497661; doi:10.3390/cimb44090283)
Supplement: Supplementary file 1 [file cimb-44-00283-s001.zip › cimb-1827255-supplementary.pdf]

Supplementary Table S1: Distribution of first-line drug-susceptibility patterns in MTB lineages.

Resistance pattern	Lineages				
	1	2	3	4	Total
HR resistant	3	7	5	1	16
HRS resistant	2	21	6	4	33
HRE resistant	1	8	3	2	14
HRSE resistant	4	56	19	13	92
HR resistant*	0	6	2	2	10
H resistant	0	1	1	0	2
S resistant	0	1	1	0	2
E resistant	0	1	0	0	1
HRSE susceptible	3	12	19	7	41
Total	13	113	56	29	211

Supplementary Table S2: Mutations found in *pncA* gene and its upstream regulatory region of the *M. tuberculosis* isolates from Nepal.

Nucleotide changes	Amino acid changes	No. of isolates	Pan-susceptible/ mono-resistant	MDR	Pre-XDR	Genotype	In vitro/in vivo susceptibility (Yadon et al., 2017)	SUSPECT-PZA	PZA susceptibility	Reported	Reference
A-11C	NA	1			1	L2	ND	NA	R	Yes	(Ramirez-Busby and Valafar, 2015)
A-11G	NA	1			1	L2	ND	NA	R/S*	Yes	(Ramirez-Busby and Valafar, 2015)
A-11G, C195T	NA, Ser65Ser	1			1	L3	ND	NA	R/S*	Yes	(Ramirez-Busby and Valafar, 2015)
ins of C at -2 and -3	NA	1		1		L1	ND	NA	S	Yes	(Ramirez-Busby and Valafar, 2015)
ins of CG at 9/10	Ala3Fs	1		1		L4	ND	NA	ND*	Novel	
T11C	Leu4Ser	6		2	4	L2	R	R	R	Yes	(Ramirez-Busby and Valafar, 2015)
T17C, C195T	Ile6Thr, Ser65Ser	1		1		L3	R	R	R	Yes	(Ramirez-Busby and Valafar, 2015)
A29C, C195T	Gln10Pro, Ser65Ser	1		1		L3	ND	R	R	Yes	(Hirano et al., 1998)
G34A	Asp12Asn	1		1		L4	R	R	R	Yes	(Sumnienski Rodrigues et al., 2005)
A35G	Asp12Gly	1		1		L1	R	R	R	Yes	(Ramirez-Busby and Valafar, 2015)
T80C	Leu27Pro	2		1	1	L2	R	R	R	Yes	(Ramirez-Busby and Valafar, 2015)
A143C	Lys48Thr	1		1		L4	R	R	R	Yes	(Ramirez-Busby and Valafar, 2015)
A152G	His51Arg	1			1	L4	R	R	R	Yes	(1999Hall1 bioedit.pdf, n.d.)
C161T	Pro54Leu	1		1		L4	R	R	R	Yes	(Ramirez-Busby and Valafar, 2015)
ins of G at 165/166	Gly55Fs	1			1	L2	ND	NA	ND*	Novel	
ins of G at 166/167	Gly56Fs	1		1		L2	ND	NA	R	Yes	(Rahman et al., 2016)
C169G	His57Asp	2		1	1	L4	R	R	R	Yes	(Ramirez-Busby and Valafar, 2015)
A170G	His57Arg	1			1	L3	R	R	R	Yes	(Ramirez-Busby and Valafar, 2015)
C176T	Ser59Phe	2		2		L4	R	R	R/S*	Yes	(Ramirez-Busby and Valafar, 2015)
C195T	Ser65Ser	37	20	15	2	L3	S	NA	R/S[a]	Yes	(Ramirez-Busby and Valafar, 2015)
T202G	Try68Gly	1		1		L2	ND	R	R	Yes	(Ramirez-Busby and Valafar, 2015)
A226C	Thr76Pro	1		1		L2	ND	R	R	Yes	(1999Hall1 bioedit.pdf, n.d.)
G233T	Gly78Val	1			1	L2	R	R	R	Yes	(Ramirez-Busby and Valafar, 2015)
T254G	Leu85Arg	3		2	1	L2	R	R	R	Yes	(Ramirez-Busby and Valafar, 2015)

G290T	Gly97Val	1			1	L2	ND	R	R	Yes	(Suzuki et al., 2002)
A298C	Thr100Pro	1		1		L4	ND	S	R	Yes	(Ramirez-Busby and Valafar, 2015)
C309G	Tyr103Stop	1		1		L4	R	NA	R	Yes	(Ramirez-Busby and Valafar, 2015)
C312G	Ser104Arg	1		1		L2	R	R	R	Yes	(Hirano et al., 1998)
G322C	Gly108Arg	2			2	L2	R	R	R	Yes	(Ramirez-Busby and Valafar, 2015)
G357C	Trp119Cys	1			1	L4	S/ND	R	R	Yes	(Ramirez-Busby and Valafar, 2015)
T359G	Leu120Arg	1		1		L2	ND	R	R	Yes	(Ramirez-Busby and Valafar, 2015)
G362C	Arg121Pro	1		1		L2	ND	R	R	Yes	(Sreevatsan et al., 1997)
del of G at 370, del of C at 372, del of T at 374, del of GATGAG from 376-381	Arg123Fs	1			1	L4	ND	NA	ND*	Novel	
del of 12 nucleotide (TGAGGTCCATGT) from 378-389	Val125Fs	1		1		L1	ND	NA	ND*	Novel	
G395C	Gly132Ala	3		2	1	L2	R	R	R	Yes	(Ramirez-Busby and Valafar, 2015)
C401T	Ala134Val	1		1		L4	R	R	R	Yes	(Ramirez-Busby and Valafar, 2015)
G406A	Asp136Asn	1			1	L4	S/R	R	R	Yes	(Ramirez-Busby and Valafar, 2015)
G406T	Asp136Tyr	1		1		L2	R	R	R	Yes	(Ramirez-Busby and Valafar, 2015)
A407G	Asp136Gly	3		3		L2	ND	R	R	Yes	(Ramirez-Busby and Valafar, 2015)
T416C	Val139Ala	2		1	1	L2	R	R	R/S*	Yes	(Hirano et al., 1998)
A424G	Thr142Ala	1			1	L2	ND	R	R	Yes	(Hirano et al., 1998)
T452C	Leu151Ser	1		1		L2	R	R	R	Yes	(Ramirez-Busby and Valafar, 2015)
C459G	Thr153Thr	1		1		L2	S	NA	ND#	Novel	
T467C	Leu156Pro	1		1		L2	R	R	R	Yes	(Ramirez-Busby and Valafar, 2015)
T470G	Val157Gly	1		1		L1	ND	S	R	Yes	(Ramirez-Busby and Valafar, 2015)
ins of G at 485/486	Gly162Fs	1		1		L4	ND	NA	ND*	Novel	
C500T	Thr167Ile	1			1	L4	ND	S	ND#	Novel	
A502C	Thr168Pro	1			1	L2	S/R	R	R*	Yes	(Ramirez-Busby and Valafar, 2015)
T545C	Leu182Ser	7		5	2	L2(6), L4(1)	R	R	R	Yes	(Lemaitre et al., 1999)
C195T, G271T	Ser65Ser, Glu91Stop	1			1	L1	R	NA	R	Yes	(Ramirez-Busby and Valafar, 2015)
C195T, A287C	Ser65Ser, Lys96Thr	2			2	L3	R	R	R	Yes	(Sumnienski Rodrigues et al., 2005)
C195T, T347C	Ser65Ser, Leu116Pro	1		1		L3	S/R	S	R/S#	Yes	(Ramirez-Busby and Valafar, 2015)
C195T, del of at 366	Ser65Ser, Arg121Fs	1		1		L3	ND	NA	R	Yes	(Hameed et al., 2020)

C195T, del of GG at 391/392	Ser65Ser, Val130Fs	1		1		L3	ND	NA	R	Yes	(Ramirez-Busby and Valafar, 2015)
C195T, ins of 524GC525	Ser65Ser, Glu174Fs	1			1	L3	ND	NA	ND*	Novel	
C195T, T545C	Ser65Ser, Leu182Ser	5		1	4	L3	R	R	R	Yes	(Lemaitre et al., 1999)
C211T, del of A at 268	His73Tyr, Ile90Fs	2		2		L1(1), L2(1)	R	R	ND*	Yes	(Ramirez-Busby and Valafar, 2015)
PCR negative		4	1		3	L1(1), L2(3)	ND	NA	R	Yes	(Suzuki et al., 2002)
WT		86	25	44	17	L1(6), L2(65), L3(4), L4(11)					
Total		211	46	108	57						

Fs: Frameshift mutation

NA: Not applicable

R: resistant

S: susceptible

ND: Not determined

*: Final interpretation as PZA resistance by combining in vitro/in vivo susceptibility, SUSPECT-PZA and literature review

[#]: Final interpretation as PZA susceptible by combining in vitro/in vivo susceptibility, SUSPECT-PZA and literature review

[a]: silent mutation associated with CAS genotype

Supplementary Table S3: Distribution of *pncA* mutation frequency among different age-wise participants.

Age-group	Number of participants	*pncA* mutation frequency
11-15	4	1 (25.0%)
16-20	21	16 (76.2%)
21-25	53	20 (37.0%)
26-30	32	16 (50%)
31-35	25	9 (36.0%)
36-40	19	9 (47.4%)
41-45	11	4 (36.4%)
46-50	9	4 (44.4%)
51-55	12	4 (33.3%)
56-60	12	3 (25.0%)
61-65	7	2 (27.5%)
66-70	3	0
71-75	1	0
76-80	1	0
81-85	1	0
